# Developing an integrated clinical decision support system for the early identification and management of kidney disease—building cross-sectoral partnerships

**DOI:** 10.1186/s12911-024-02471-w

**Published:** 2024-03-08

**Authors:** Gillian Gorham, Asanga Abeyaratne, Sam Heard, Liz Moore, Pratish George, Paul Kamler, Sandawana William Majoni, Winnie Chen, Bhavya Balasubramanya, Mohammad Radwanur Talukder, Sophie Pascoe, Adam Whitehead, Cherian Sajiv, Louise Maple-Brown, Nadarajah Kangaharan, Alan Cass

**Affiliations:** 1 Wellbeing and Preventable Chronic Diseases Division, Menzies School of Health Research, Charles Darwin University, PO Box 41096, Darwin, NT 0810 Australia; 2 Department of Nephrology, Royal Darwin Hospital, Northern Territory Health, Darwin, NT Australia; 3 Central Australian Aboriginal Congress, Aboriginal Corporation, Alice Springs, NT Australia; 4 Aboriginal Medical Services Alliance Northern Territory, Darwin, NT Australia; 5 Department of Nephrology, Alice Springs Hospital, Northern Territory Health, Alice Springs, NT Australia; 6 Northern Territory Medical Program, Flinders University, Royal Darwin Hospital Campus, Darwin, NT Australia; 7 Radical Systems, Darwin, NT Australia; 8 Department of Endocrinology, Royal Darwin Hospital Northern Territory Health, Darwin, NT Australia; 9 Division of Medicine, Royal Darwin Hospital Northern Territory Health, Darwin, NT Australia

**Keywords:** Integrated patient care, Clinical safety, Clinical information systems, Chronic disease, Clinical decision support, Clinical algorithms, Remote health

## Abstract

**Background:**

The burden of chronic conditions is growing in Australia with people in remote areas experiencing high rates of disease, especially kidney disease. Health care in remote areas of the Northern Territory (NT) is complicated by a mobile population, high staff turnover, poor communication between health services and complex comorbid health conditions requiring multidisciplinary care.

**Aim:**

This paper aims to describe the collaborative process between research, government and non-government health services to develop an integrated clinical decision support system to improve patient care.

**Methods:**

Building on established partnerships in the government and Aboriginal Community-Controlled Health Service (ACCHS) sectors, we developed a novel digital clinical decision support system for people at risk of developing kidney disease (due to hypertension, diabetes, cardiovascular disease) or with kidney disease. A cross-organisational and multidisciplinary Steering Committee has overseen the design, development and implementation stages. Further, the system’s design and functionality were strongly informed by experts (Clinical Reference Group and Technical Working Group), health service providers, and end-user feedback through a formative evaluation.

**Results:**

We established data sharing agreements with 11 ACCHS to link patient level data with 56 government primary health services and six hospitals. Electronic Health Record (EHR) data, based on agreed criteria, is automatically and securely transferred from 15 existing EHR platforms. Through clinician-determined algorithms, the system assists clinicians to diagnose, monitor and provide guideline-based care for individuals, as well as service-level risk stratification and alerts for clinically significant events.

**Conclusion:**

Disconnected health services and separate EHRs result in information gaps and a health and safety risk, particularly for patients who access multiple health services. However, barriers to clinical data sharing between health services still exist. In this first phase, we report how robust partnerships and effective governance processes can overcome these barriers to support clinical decision making and contribute to holistic care.

## Background

Chronic conditions, including the interrelated conditions of chronic kidney disease (CKD), diabetes and cardiovascular disease, account for high mortality and morbidity globally and Australia wide [1]. The Grattan Institute notes that, at best, Australia’s primary care system provides only half of the guideline-recommended care for many chronic conditions. Despite significant funding allocated to support the planning, coordination and management of chronic conditions in primary care [2], the number of potentially preventable hospital admissions across Australia remains high. Evidence suggests that sharing patient clinical information across health services can decrease preventable hospital admissions by improving communication and coordination between healthcare providers [3, 4].

The burden of chronic conditions is particularly high in the Northern Territory (NT) of Australia, where 30% of the population are First Nations Australians. Multi-morbidity is common with approximately 60% of Territorians over the age of 50 living with at least two chronic conditions [5]. Moreover, the NT has the highest rates of severe or end-stage kidney disease in Australia, with a relentless increase in the numbers of patients requiring kidney replacement therapy (KRT) exceeding repeat demand projections made by NT Health [6]. Services continue to be stretched beyond their capacity and the economic burden on the public health system is significant [7]. Critically, little is known of the community burden of CKD, as there is no central database or CKD registry in Australia.

Local studies have found that many primary health services were caring for patients with advanced kidney disease, without the support of tertiary nephrology services [8]. The rise in complex conditions and difficulties delivering services within budgetary constraints, particularly in remote areas, placed many services under additional stress [9]. In 2014, our research team was invited by an Aboriginal Community-Controlled Health Service (ACCHS) to undertake a longitudinal analysis of their CKD management program [10]. This involved a retrospective analysis of patient level data for screening, identification and management of CKD over the previous 10 years. As all ACCHS in the NT use the same proprietary Electronic Health Record (EHR), we were able to apply the same data extraction script and offer a similar longitudinal analysis to other ACCHS. The individual and combined findings highlighted a significant increase in the number of people identified with a chronic condition over the previous 10 years, along with improvements in the attainment of clinical targets from baseline. However, it also noted that annual screening of people with significant risk factors (diabetes, cardiovascular disease, hypertension) was suboptimal and the accurate diagnosis of CKD categories 3b, 4 and 5, based on pathology results, could be improved to facilitate appropriate guideline-based management and treatment plans.

The findings aligned with a recent systematic review of effective CKD programs for First Nations patients [11]. This review highlighted that CKD management was improved with programs that focussed on regular screening of people at risk of CKD, early intervention, timely referral to specialist services and adherence to evidence-based guidelines. Importantly, supporting and embedding care in primary health services was key to patient engagement and uptake of services [11].

Despite efforts to improve sharing of patient information across the primary-tertiary interface in the NT, challenges remained in the identification and management of complex conditions. These included multiple patients records across services; incomplete or conflicting data from different service providers; and the time and cognitive burden placed on clinicians to gather, synthesise and interpret the data. Coupled with strained budgets, high staff turnover [12] and a highly mobile population where people may access multiple community and hospital-based services, service delivery for people living with complex chronic disease is both challenging and complicated.

Australia’s health system is described as a hybrid model. It consists of publicly-funded health services based on the premise of universal access to health care, and privately-funded services based on user choice [13]. Most health services (primary and tertiary) in the NT are publicly funded, particularly those in rural and remote locations.

Service providers and clinicians recognise the need for a focus on integrated care [14] and a move away from management of separate conditions in isolation [15]. Patients also want systems of care that improve their journey, support their general practitioners (GPs) to provide holistic care and, for those with complex conditions, reduce the number of specialists they see (specialist fatigue) [16]. Integrated models of care that focus on prevention and care coordination can slow the progression of chronic conditions, reducing complications of concomitant conditions resulting in substantial health, social and economic benefits [17].

### Objectives

This paper describes the methods, challenges and lessons learned from the collaborative co-design process and implementation of Territory Kidney Care (TKC).

The TKC initiative was established to address the disconnect between primary and tertiary, government and non-government health services in the early identification and management of CKD in the NT. The aim was to improve the integration of health data, facilitate efficiencies in data collation and analysis, and implement evidence-based guidelines to enhance the patient journey and reduce the burden of CKD in the NT.

As a clinical decision support system, TKC is intended to:

- Improve the quality and comprehensiveness of data available to all clinicians providing care to a patient.
- Create efficiencies in workflows through automated identification and summation.
- Assist clinicians particularly remote-based clinicians with risk stratification and earlier specialist support for the management of complex conditions.
- Decrease the rates of unplanned hospital admissions related to kidney disease through proactive identification and intervention.
- Address the escalating demand for kidney treatments in the NT.

Through our established relationships with many ACCHS and NT Health, we proposed the TKC initiative to reduce the burden of CKD to the Federal and NT Health Ministers and the peak Aboriginal health representative body in the NT, Aboriginal Medical Services Alliance Northern Territory (AMSANT). Menzies School of Health Research (Menzies) received in-principal support from stakeholders to pursue funding to develop an integrated clinical information system.

## Method and materials

A systematic review of barriers and facilitators to the uptake of clinical decision support (CDS) systems informed the design and development of TKC [18, 19]. Our review of CDS designs found that chronic disease CDS is often single-disease focused, and rarely incorporates sufficient EHR data to be applicable in multimorbidity. Furthermore, up to 80% of user interfaces, focused on alerts and reminders, which are associated with alert fatigue and overriding behaviours [18]. To improve on the design of previous CDS tools, the approach TKC takes to communicating clinical decision support is primarily via an automated patient summary, similar to the problems list of a physician letter or discharge summary. The meta-aggregation from our qualitative review, found that CDS uptake is dependent on clinical context, user, external context and technical factors [19]. Previous implementation science frameworks have also described a similarly broad number of factors to be considered in CDS implementation [20, 21]. TKC implementation proactively addressed these complex factors - for example through clinical champions, strong user engagement in the design, implementation officers, as well as planning for long term funding to sustain ongoing implementation and system development to address external context barriers to uptake. TKC was designed to be a value-add proposition for clinicians and health services to improve the care of patients diagnosed with, or at risk of, kidney disease. The main goal is to provide clinicians with access to real-time, consolidated, intelligently presented, longitudinal EHR data to close the information gap and reduce cognitive load [22]. Additionally, we envisage that TKC will provide some functions of a registry, for example, outcomes against targets for service and population-level monitoring and continuous quality improvement (CQI), activity and cost data for annual reporting and advocacy. TKC is an adjunct to clinical information systems in partnering health services and therefore does not require clinicians to adopt a new EHR, train in data entry or involve duplicate documentation. From an operational perspective, it is a non-critical system and, hence, does not require 24-hour maintenance and support.

### Governance

TKC had a strong foundation as the business case was fully supported by health services on the ground and by their overarching organisations, including at the ministerial level. In 2017, partners and stakeholders undertook an initial workshop to determine the scope of the system, identify benefits, limitations and potential barriers. The sovereignty of patient data from ACCHSs was recognised [23], with stakeholders agreeing that operational and technical requirements must protect this right into the future. Data security was of utmost importance to all stakeholders. As such, it was agreed the system would sit within the NT Government’s (NTG) infrastructure, adopting best practice Information and Communication Technology (ICT) security protocols.

A governance structure that oversaw TKC from the design phase to the business-as-usual phase included an overarching Steering Committee, Clinical Reference Group (CRG), Technical Working Group (TWG) and project management team. Membership of the Steering Committee was broad and representative of stakeholders involved in the planning and delivery of primary and tertiary care services in the NT. Members included representatives from: the NTG data warehouses; ICT and clinical information systems; the governing bodies for ACCHS (AMSANT); private GP practices (NT Primary Health Network); GPs from ACCHS; and clinicians from primary and tertiary sectors of the government. During implementation there was stronger representation from ACCHS (clinicians and Aboriginal Health Practitioners) and the NTG’s clinical information and systems integration divisions.

Risk management of clinical hazards related to the design, development and deployment of TKC, was guided by the Western Australian Government’s Department of Health ‘ICT Patient Safety Risk Assessment: Guide for ICT Projects’ and the United Kingdom’s National Health Service (NHS) ‘Clinical Risk Management Guidelines’ [24, 25]. Ongoing advice from the Executive Steering Committee, the CRG and TWG informed the build of a prototype, which was critical in identifying required controls and risk mitigation strategies in a Clinical Risk Management Plan. The full system build deployed these controls, which were subsequently tested and validated with stakeholders to ensure the system continued to meet clinician and industry standards.

### Design

Based on the intent of the system and the problems to be addressed, partners agreed on the inclusion/exclusion criteria for the collection of patient data and data sharing. Over the next two years, independent legal advice with a focus on privacy and confidentiality, informed the consent model (including ability to opt-out at the individual and health service level) and data sharing agreements. Table 1 below outlines the data components that define the inclusion/exclusion criteria for TKC eligible patients - that is, people at risk of kidney disease or living with kidney disease. Subsequent EHR data extracted for eligible individuals are comprehensive and include historical information. Components include, but are not limited to, patient identifiers, demographics, diagnosis and procedure codes, medications, observations, laboratory results, and radiology data [26].

**Table 1:** Exclusion/inclusion criteria and data components for patients included in TKC

Exclusion Criteria	Components
**Patient is not fictious, has not opted out of TKC, is not deceased and is ‘Current’**	Date of death; Opt-out status; active and current status within electronic health record
Inclusion Criteria	Components
**Patient 16 years or older at time of data extraction**	Date of birth
**Has kidney disease: coded data**	ICPC: U59 Dialysis; U28 Renal Transplant; U88 Glomerulonephritis/nephrosis; U99 Urinary disease; U28 Limited urinary function ICD 10-AM: Z49.1-Dialysis; T85.71 Infection peritoneal dialysis catheter; T86.1 Renal transplant rejection; Z49% Dialysis; Z94.0 Kidney transplant
**Has significant risk factors for kidney disease: coded data**	ICPC: K87 Hypertension complicated; T89 Diabetes insulin dependent; T90 Diabetes non-insulin dependent ICD 10-AM: E10% Type 1 diabetes; E11% Type 2 Diabetes; I00-I99 Diseases of the circulatory system; N00-N08 Glomerular disease; N10-N16 Renal tubule interstitial disease; N17-N19 Kidney failure; N20-N23 Renal calculi; N24-N29 Disorders of kidney and ureter; Q60-Q64 Congenital kidney malformations
**Measurements used in calculations to determine ‘Risk’**	HbA1c (%) measure of over 6.5% eGFR measure (if present) < 90 ACR result > 2.5 BMI > 30 Cardiovascular Risk Score > 15

Legend: ICPC – International Classification of Primary Care; ICD 10-AM – International Statistical Classification of Diseases 10th edition Australian Modification

We followed an incremental software development model [27] that included: requirement gathering, design, development, validation and testing, requirements adjustment, knowledge acquisition, knowledge engineering, validation and testing, deployment. The TWG provided advice and determined solutions to issues including automating extracts from health services; secure system-to-system data transfer; opt-out functionality; and secure messaging between government and non-government EHR. The CRG determined and validated algorithms for diagnosis, prognosis, risk stratification and clinically significant event alerts [26]. Clinicians included GPs, nephrologists, endocrinologists, cardiologists, and nursing specialists. All clinical algorithms were underpinned by an evidence base, with guidelines/references included in TKC and easily accessible to system users. The subject matter experts recommended guidelines. For example, cardiologists determined the guidelines to be used for diagnosis and management of hypertension. The subject matter experts also advised when algorithms needed to be updated due to changes in guidelines. Examples of guidelines used to inform diagnostic algorithms include 2012 KDIGO CKD Guidelines [28] and Heart Foundation Guidelines for Diagnosis and Management of Hypertension 2016 [29]. A GP and nephrologist tested and validated all algorithms in the development environment. A formal validation study demonstrated high accuracy of key clinical algorithms [26]. The CRG was instrumental in ensuring system capabilities were clinician focused and that the interface was user friendly, intuitive and navigable for time poor clinicians.

**Fig. 1:**
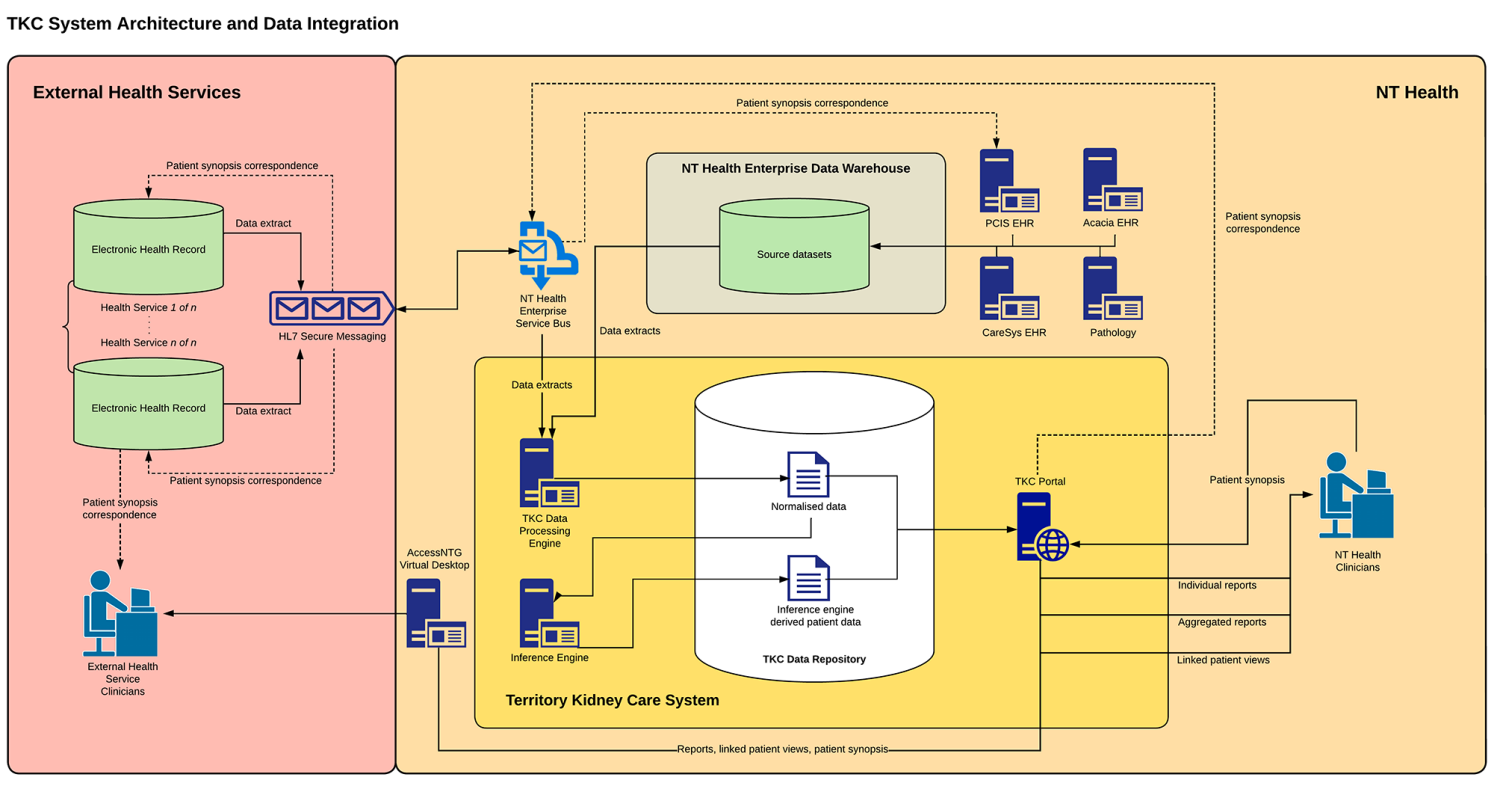
System architecture diagram

Data structures were determined, with three environments (development, testing and live) established on a standalone server within the NT Health’s data warehouse. In the initial stages, only NT Health data entered the TKC system with a weekly refresh of real-time data from the warehouse sent to the development environment to enable clinicians on the team to test and validate the accuracy of the algorithms. As the system sat on a government server, changes and integration updates were subject to the NTG’s Change Release Protocols. All releases followed the NTG’s change management processes and were approved by the relevant Department: testing methodologies included system integration testing; user acceptance testing in the test environment; and product verification testing. For automated extracts to be securely transferred from external sources through the NTG firewalls to TKC, a secure interface was necessary. This was simplified as all ACCHSs in the NT use the same vendor for their EHR. Menzies commissioned the vendor to develop an application programming interface (API) that was made available to health services in the next release. End-to-end testing of the secure transfer of data extracts was undertaken alongside testing of the secure delivery of clinical messages from TKC to patient records in government and non-government clinical systems. The system architecture diagram in Fig. 1 describes the data flow (Fig. 1).

#### Development

Once the prototype was developed, Memoranda of Understanding were entered into with four ACCHSs to share a single data extract with TKC for the purposes of validation. This process was necessary to prepare for the implementation phase and the inclusion of data that was not already harmonized. Data was deleted once mapping had been completed. Considerable effort was required to map and standardise clinical components (for example, laboratory measurements, physical results and medications) from different EHRs and laboratory providers. The addition of non-government patient records presented challenges for the linkage of patient data across multiple services without the presence of a unique identifier [30]. Due to the reality of multiple aliases (names, date of birth) for many patients in the NT, the TKC linking algorithm is weighted to not link records unless all criteria are met. External testing of the accuracy of the patient record linkage protocol found 97% accuracy with 3% of records identified as false negatives (likely the records belonged to the same patient but were not linked) and 0% false positives (patient records did not belong to the same patient). Administrator protocols include audits of linkages enabling data cleaning (multiple aliases, mis-entered or missing unique identifiers) and updating of records at the health service level.

### Implementation

Each ACCHS sought approval from their governing Board to participate in TKC, which was formalised through a Data Participation Agreement (DPA). The DPA describes the storage, security, ownership of data as well as the *agreed use cases* for the data. Agreed use cases were categorised into three levels: (1) sharing of identified and individual patient information for clinical care delivery; (2) sharing of lists of identifiable patients (with or without clinical data) meeting a condition for monitoring or planning of clinical care (e.g. advanced CKD without recommended medication); (3) de-identified aggregated reports for CQI, evaluation, advocacy and annual reporting.

Implementation plans were individualized for each participating health service and included a range of requirements, such as: technical enablers within source systems; training, permissions/access; destination for CQI reports, patient advice; patient privacy notices and facilitation of opt-out. All activities in the implementation plan were supported by the Menzies project team, including demonstrations to governing Boards and clinicians, assisting with technical changes, development of health service specific patient information (posters, flyers, videos), often translated into Aboriginal languages to meet local needs. These plans were designed to identify and address potential implementation issues at each site.

Once all activities in the implementation plan were completed, an initial extract from each ACCHS was sent to TKC for validation. This allowed the ACCHS to check the data extracted, confirm the inclusion/exclusion criteria and make changes if necessary. It also allowed the project team to check for health service specific coding or data anomalies that would require additional mapping. Although all the ACCHSs have the same proprietary EHR, each health service is independent and may describe conditions (uncoded data) differently or use a variety of pathology providers with varying units of measurements. The initial extract also provided an opportunity to support CQI activities within the ACCHS by identifying the number of patient records unable to be linked due to missing or incorrect identifiers.

### Evaluation

Each partnering health service acknowledges the importance of a robust evaluation, as outlined in the DPA, that includes formative, process and summative components. The qualitative components of the formative evaluation included interviews, surveys, workshops and focus groups with health service managers, implementation officers and clinical end users at each site, to ensure the design and user interface met end user’s needs (unpublished). Formal testing of the algorithms ensured the system was fit for purpose [26]. A mixed methods evaluation commenced in 2022 and quantitative and qualitative data from interviews at baseline and post implementation, focus groups and user feedback are currently being analysed. This process evaluation has helped identify change processes to improve the uptake at subsequent sites and system requirements for improved effectiveness and efficiency of TKC. A summative evaluation is scheduled for 2025 and will include quantitative and economic data to assess the impact of TKC, including the costs and benefits of the system from a pre-intervention baseline in 2017.

### Findings

**Fig. 2:**
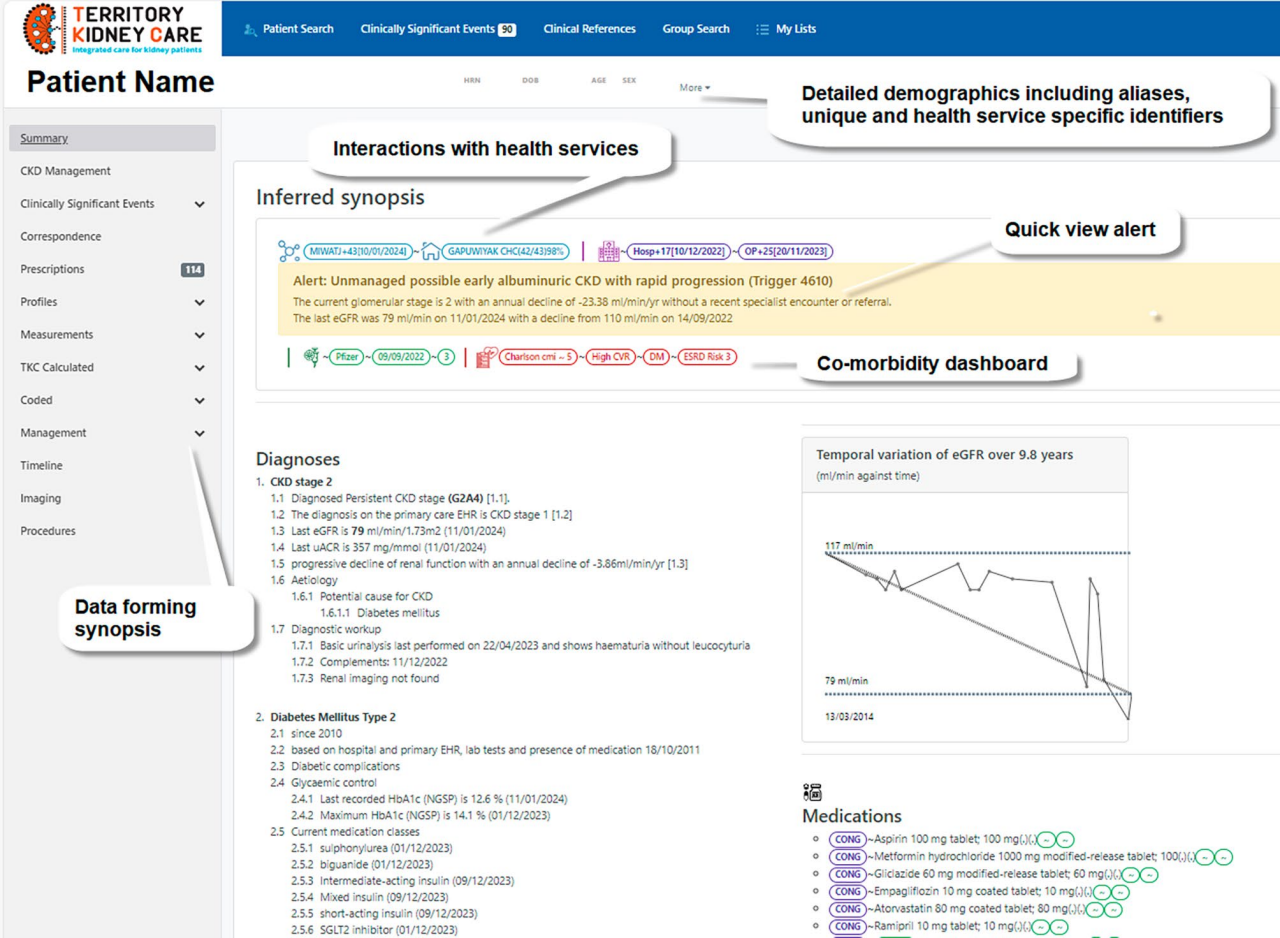
Extract of a patient synopsis report

At the time of writing, 56 government primary health services, 6 government hospitals and 11 out of the 13 ACCHS in the NT have DPAs in place and are participating in TKC. TKC currently brings together data on nearly 69,000 individuals who have risk factors for or have kidney disease. The functionality offered by TKC has been described by clinicians as a ‘game changer’. Clinicians in primary health have noted the benefits of the patient synopsis function (Fig. 2) for immediate summation of longitudinal data, which reduces time required to collate, analyse and synthesise information from multiple sources. The patient synopsis presents data intelligently, reflecting clinician thought processes and outlining diagnosis dates and last episodes of care, significantly reducing cognitive load and clinician fatigue [31]. Demographics identify places of encounter if they have attended a TKC participating health service. GPs have noted the ease of navigation, the comprehensiveness of data and the benefits for managing complex conditions. Potentially avoidable medical events are identified through clinically defined algorithms that constantly surveille incoming data. The *Clinically Significant Events* tab facilitates pre-emptive care by alerting nephrologists to potential untoward events for individuals. Early analysis has shown 15% of people with advanced CKD (CKD3b-5) do not have a corresponding diagnosis in the EHR. As such, clinicians can use TKC to prepare clinic lists, based on CKD category, to regularly identify undiagnosed cases of CKD, allowing early referral [32] and appropriate management plans to be implemented.

TKC has been designed by clinicians for clinicians, with a strong focus on the requirements of primary health. Importantly, key members of the project and technical team also have medical backgrounds, ensuring the translation of requirements considered the user experience of the clinician foremost. A mixed methods evaluation has commenced with a process evaluation assessing the design and implementation of TKC. A summative evaluation will assess the impact of TKC including the costs and benefits of the system from a baseline in 2017.

## Discussion

**Fig. 3:**
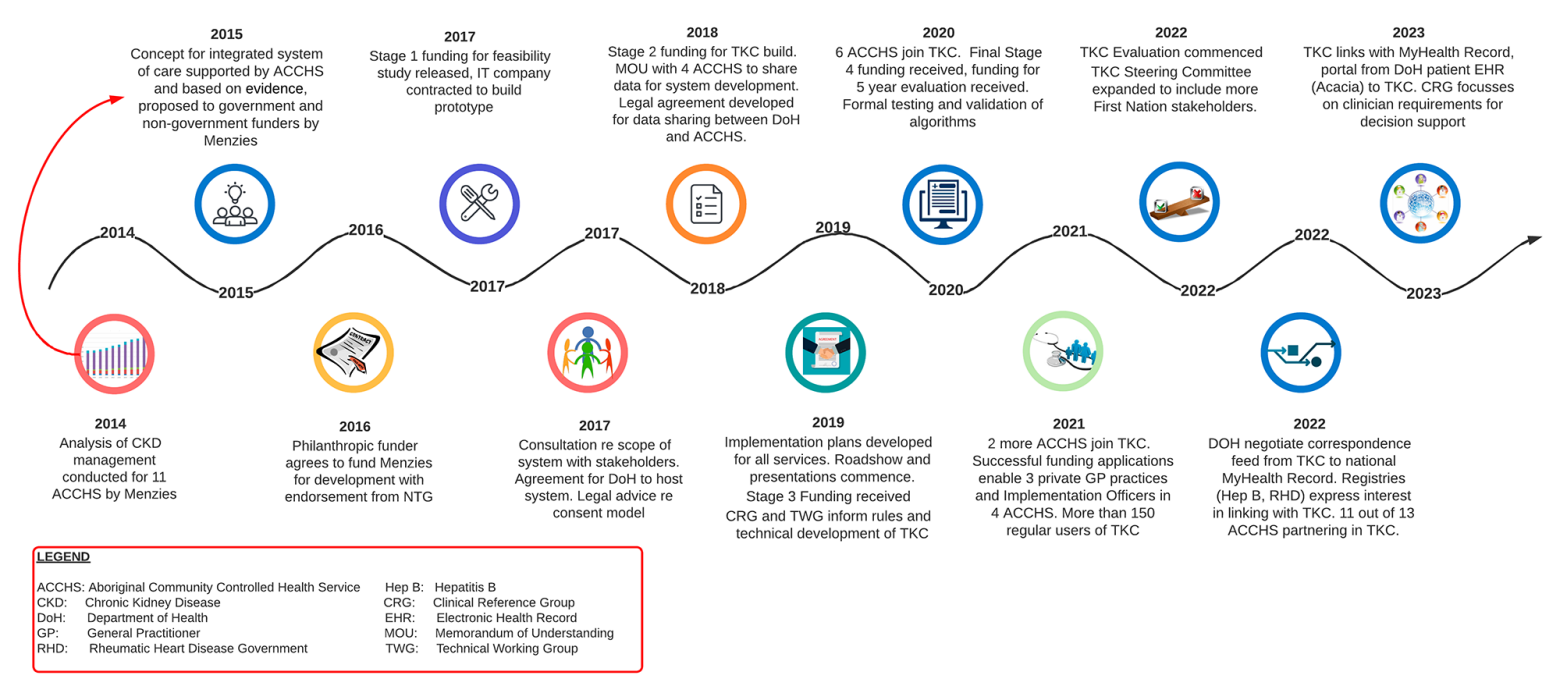
TKC development timeline

The ongoing success of TKC is dependent on support and trusting relationships between stakeholders and partners as well as a shared vision for improving patient care. EHR data is rich and has great potential for use to improve clinical care and population health. However, optimising use of EHR data requires overcoming both technical issues, for example interoperability, as well as ethical and legal issues around data sharing. Our learnings included the need for frequent and digestible communiques and opportunities to request further information, flexible timelines, individualised implementation strategies and patience. As such our development took an agile approach that was reflected in contracts with our funder and digital contractor, and acknowledged the new ground we were traversing. In TKC, partnering health services weighed up the benefits and risks of sharing clinical data of their patient cohorts and were satisfied with the governance processes put in place for the primary and secondary use of data. The establishment of DPAs and implementation plans was paced according to the partnering health services’ readiness, and, while this did not meet the timing of initial milestones of our funder, our flexible approach yielded greater benefits in terms of engagement and uptake (Fig. 3). This was an important aspect of our initiative.

While driven and developed by a research institute (Menzies), TKC is not a research project in the traditional sense. It uses principles and methodologies of implementation science to address identified evidence-practice gaps and to improve health systems. However, we have strived to adhere to principles of ethical research with Aboriginal peoples and communities ensuring there has been strong engagement, governance and capacity building in partnership with ACCHS. The design of TKC was determined by the health needs of Aboriginal people and communities and informed by previous work conducted with ACCHS. DPAs and implementation plans were individualised by ACCHS according to their requirements and approval of their respective Boards. The Executive Steering Committee has strong representation from ACCHS and their governing body AMSANT. Further, we have supported capacity building within ACCHS by funding implementation officers in four health services to advise on uptake, issues and functional requirements of TKC.

### Sustainability

In terms of sustainability, TKC will continue to engage stakeholders through the Executive Steering Committee, CRG and demonstrations. TKC has been endorsed by the Australian Digital Health Agency with recent work connecting TKC to the national My Health Record so that government clinicians may share specialist letters with the site. In addition, TKC has been embedded into ACACIA, the new EHR being implemented across NT Health, demonstrating it is a government endorsed and supported system. This will also enable easier access to a patient’s EHR in TKC for NTG clinicians. We have worked with NTG and ACCHS to improve the process for nongovernment clinicians to access TKC via the secure, external NTG portal, reducing the total number of clicks from 16 to four. This will undoubtedly reduce ACHHS clinician frustration and improve uptake.

### Significance

Linking and integrating patient health data across different systems and providers is essential for providing comprehensive and safe care, particularly for people utilising multiple health services [33]. In the NT, a large proportion of the population are highly mobile, visit multiple health services and have complex health and social needs. They often face challenges in accessing timely and appropriate health care due to barriers related to language, transport, unstable accommodation, or discrimination. Primary health services may also have difficulty in delivering quality care to the highly mobile population, due to limited funding, increasing demand, workforce shortages, lack of infrastructure and inadequate data systems. Integrating and summarising EHR data within TKC will provide comprehensive information coverage across the whole of the NT for the first time. It will allow health professionals to have a complete and accurate picture of a patient’s medical history, current conditions, medications, immunizations and other information. TKC is primarily clinician facing but the synopsis report and related education material within the platform, are useful for shared use during patient consults. TKC can improve the safety, quality and cost-effectiveness of clinical care by flagging high clinical risk patients, pre-empting adverse events, avoiding duplication of tests or treatments, and facilitating coordination and continuity of care. TKC data can also be used to improve the understanding of resource demand by analyzing health needs, attendance patterns and outcomes. This can help identify gaps and opportunities for improving the quality and accessibility of primary health care, as well as inform policy making and resource allocation.

## Conclusion

TKC was borne out of necessity and is a product of clinician and patient needs to find a new way of addressing the growing demand for renal services and understand the true burden of kidney disease in the NT. The business case and design of TKC was informed by collaborative partnerships with ACCHSs and NT Health and extensive research undertaken by Menzies over many years. Investigations included a review of cost-effective CKD management programs; economic benefits of clinical data registries; and barriers/facilitators to the uptake of technologies. The design and development of TKC has been an iterative process with extensive user engagement and input to ensure the system supported and enhanced the care delivered. TKC embodies evidence translation to address challenges in health service delivery in the NT.

## Data Availability

The data that supports the findings are owned by the participating services and requires their individual permission for release. However, data are available from the authors on request and with the permission of the TKC Steering Committee and individual health service.

## Abbreviations

ACCHS: Aboriginal Community Controlled Health Service
AMSANT: Aboriginal Medical Services Alliance Northern Territory
API: Application Programming Interface
CDS: Clinical Decision Support
CKD: Chronic Kidney Disease
CRG: Clinical Reference Group
CQI: Continuous Quality Improvement
DPA: Data Participation Agreement
EHR: Electronic Health Record
ICD 10AM: International Statistical Classification of Diseases 10th edition Australian Modification
ICT: Information and Communication Technology
ICPC: International Classification of Primary Care
KRT: Kidney Replacement Therapy
PCIS: Primary Care Information System
NHS: National Health Service
NT: Northern Territory
NTG: Northern Territory Government
TKC: Territory Kidney Care
TWG: Technical Working Group
