# Correction: Developing an integrated clinical decision support system for the early identification and management of kidney disease—building cross-sectoral partnerships

**DOI:** 10.1186/s12911-024-02492-5

**Published:** 2024-03-28

**Authors:** Gillian Gorham, Asanga Abeyaratne, Sam Heard, Liz Moore, Pratish George, Paul Kamler, Sandawana William Majoni, Winnie Chen, Bhavya Balasubramanya, Mohammad Radwanur Talukder, Sophie Pascoe, Adam Whitehead, Cherian Sajiv, Louise Maple-Brown, Nadarajah Kangaharan, Alan Cass

**Affiliations:** 1 Wellbeing and Preventable Chronic Diseases Division, Menzies School of Health Research, Charles Darwin University, 0810 Darwin, NT PO Box 41096, Australia; 2 Department of Nephrology, Royal Darwin Hospital, Northern Territory Health, Darwin, NT Australia; 3 Central Australian Aboriginal Congress, Aboriginal Corporation, Alice Springs, NT Australia; 4 Aboriginal Medical Services Alliance Northern Territory, Darwin, NT Australia; 5 Department of Nephrology, Alice Springs Hospital, Northern Territory Health, Alice Springs, NT Australia; 6 Northern Territory Medical Program, Flinders University, Royal Darwin Hospital Campus, Darwin, NT Australia; 7 Radical Systems, Darwin, NT Australia; 8 Department of Endocrinology, Royal Darwin Hospital Northern Territory Health, Darwin, NT Australia; 9 Division of Medicine, Royal Darwin Hospital Northern Territory Health, Darwin, NT Australia

**Correction: Gorham et al. BMC Medical Informatics and Decision Making (2024) 24:69**

10.1186/s12911-024-02471-w

In the original article, a hyphen was omitted from the surname of author Louise Maple-Brown. The spelling has been corrected, as shown in this Correction article.

The original article [1] has been corrected.
